# Novel Machine-Learning Based Framework Using Electroretinography Data for the Detection of Early-Stage Glaucoma

**DOI:** 10.3389/fnins.2022.869137

**Published:** 2022-05-04

**Authors:** Mohan Kumar Gajendran, Landon J. Rohowetz, Peter Koulen, Amirfarhang Mehdizadeh

**Affiliations:** ^1^Department of Civil and Mechanical Engineering, School of Computing and Engineering, University of Missouri-Kansas City, Kansas City, MO, United States; ^2^Vision Research Center, Department of Ophthalmology, University of Missouri-Kansas City, Kansas City, MO, United States; ^3^Department of Biomedical Sciences, University of Missouri-Kansas City, Kansas City, MO, United States

**Keywords:** glaucoma, machine learning, electroretinography, ERG, wavelet transform, early stage, AI

## Abstract

**Purpose:**

Early-stage glaucoma diagnosis has been a challenging problem in ophthalmology. The current state-of-the-art glaucoma diagnosis techniques do not completely leverage the functional measures' such as electroretinogram's immense potential; instead, focus is on structural measures like optical coherence tomography. The current study aims to take a foundational step toward the development of a novel and reliable predictive framework for early detection of glaucoma using machine-learning-based algorithm capable of leveraging medically relevant information that ERG signals contain.

**Methods:**

ERG signals from 60 eyes of DBA/2 mice were grouped for binary classification based on age. The signals were also grouped based on intraocular pressure (IOP) for multiclass classification. Statistical and wavelet-based features were engineered and extracted. Important predictors (ERG tests and features) were determined, and the performance of five machine learning-based methods were evaluated.

**Results:**

Random forest (bagged trees) ensemble classifier provided the best performance in both binary and multiclass classification of ERG signals. An accuracy of 91.7 and 80% was achieved for binary and multiclass classification, respectively, suggesting that machine-learning-based models can detect subtle changes in ERG signals if trained using advanced features such as those based on wavelet analyses.

**Conclusions:**

The present study describes a novel, machine-learning-based method to analyze ERG signals providing additional information that may be used to detect early-stage glaucoma. Based on promising performance metrics obtained using the proposed machine-learning-based framework leveraging an established ERG data set, we conclude that the novel framework allows for detection of functional deficits of early/various stages of glaucoma in mice.

## 1. Introduction

Glaucoma, a chronic neurodegenerative disease affecting the retina and optic nerve, and a leading cause of blindness, is characterized by a progressive, irreversible loss of vision. As currently available treatment paradigms focus primarily on a predisposing factor, elevated intraocular pressure (IOP), and do not allow for repair of the retina and optic nerve once the disease has progressed and damage has occurred, technologies enabling an early diagnosis of glaucoma are needed urgently. Consequently, such new diagnostic modalities enabling early therapeutic intervention would significantly improve treatment outcomes. Current methods of glaucoma diagnosis are based on psychophysical techniques and the assessment of structural changes to the retina and optic nerve (Bussel et al., 2014). Standard automated perimetry testing, including the widely used Humphrey visual field testing, currently represents the most commonly utilized technique for glaucoma diagnosis and monitoring of disease progression and therapy outcomes (Ernest et al., 2012; Fidalgo et al., 2015). Recent efforts to employ machine-learning (ML) approaches to improve the analysis of behavioral psychophysical testing approaches produced moderate improvements over conventional analysis algorithms (Saeedi et al., 2021). However, significant damage to the retina and optic nerve, including loss of retinal ganglion cells (RGCs) has often already occurred before changes can be detected with standard automated perimetry testing (Turalba and Grosskreutz, 2010).

Recently, automated retinal image analysis (ARIA) systems have been developed for the diagnosis of complex diseases such as diabetic retinopathy and glaucoma (Sim et al., 2015; Lee et al., 2017). The development of these ARIA systems involved ML-based methods to detect structural changes determined with optical coherence tomography (OCT) imaging resulting in high analytical accuracy in automatically classifying disease phenotypes based on structural characteristics (Zhu et al., 2014; Asaoka et al., 2016; An et al., 2019). Despite such significant progress, early detection of glaucoma is still a challenge (Brandao et al., 2017), given the highly significant limitations of early detection of glaucoma based on structural methods. Systems employing the analysis of structural changes for glaucoma diagnosis are based on measuring retinal nerve fiber layer (RNFL) thickness in OCT images of the retina, which is highly variable and weakly correlated with RGC counts despite RNFL thickness being a surrogate marker of RGC degeneration and optic nerve fiber loss, hallmarks of glaucoma pathogenesis (Ledolter et al., 2015). Further, RGC loss often occurs early during pathogenesis in the absence of measurable RNFL thinning, prompting an urgent clinical need for methods with higher sensitivity, such as functional measures including ERG (Harwerth et al., 2002; Fortune et al., 2003; Takagi et al., 2012; Ledolter et al., 2015; Brandao et al., 2017). In contrast, functional measures such as visual field and ERG are sensitive to subtle changes in RGC function and RGC damage, which suggest a significant potential to enable early detection of glaucoma, even in the absence of elevated IOP, as seen in patients with normotensive glaucoma (Fortune et al., 2003; Aldebasi et al., 2004; Brandao et al., 2017). Therefore, this study aims to investigate such potential considering ERG signals.

Consequently, interventions could be initiated before irreversible damage occurs, allowing for the optimization of treatment strategies based on the improvement of RGC function (Ventura and Porciatti, 2006). This is of high clinical importance as determining the efficacy of therapies aimed at lowering IOP in open-angle glaucoma (Palmberg, 2002; Leske et al., 2007) requires early validation of therapy success (An et al., 2019), but will also be of importance for the development of novel alternative and complementary glaucoma therapies based on neuroprotective strategies (Rohowetz et al., 2018). Recently, in a study conducted by Tang et al. (2020) photopic negative response (PhNR) was used to assess the short-term changes in inner retinal function following intraocular pressure (IOP) decrease in glaucoma using eyedrops. Hui et al. (2020) showed that Nicotinamide supplementation helps improve the function of the inner retina in glaucoma.

Recent advances in the acquisition of complex neuroscience data have a significant innovative potential to progress toward more effective diagnostic systems (Kononenko, 2001). The adequate, timely, and clinically relevant analysis of such data has potentially high clinical impact (Lisboa, 2002). However, while such data sets can be readily acquired and technologies to further improve and simplify data acquisition continue to emerge (McPadden et al., 2019), critical barriers to implement the effective use of such novel data in clinical diagnostics and therapy delivery remain (Lee and Yoon, 2017). While the analysis of complex biomedical data is often part of medical diagnostics, current expert analysis standards and algorithms are limited by pattern recognition in few dimensions, which results in less than optimal identification or even exclusion of potentially relevant diagnostic features (Hannun et al., 2019). Machine learning could significantly augment medical diagnostics and increase their efficacy by analyzing aspects of complex and multi-dimensional biomedical data that are either not being considered adequately or that are not accessible to current analysis methods (Holzinger, 2014). Such machine-learning based diagnostic approaches have been developed and are being actively used for the detection of cardiovascular diseases (Al'Aref et al., 2019), and cancer (Cruz and Wishart, 2006).

ERG data are one such type of complex and multi-dimensional biomedical data that are potentially relevant to the diagnosis of glaucoma, but are currently not considered during routine clinical practice or in clinical research. Historically, this is due to multiple barriers related to clinical ERG data acquisition, such as limitations in reproducibility, high costs of both equipment and of individual tests, long test duration and complex test administration resulting in reduced patient acceptance and compliance, and the need for highly trained experts to administer tests. With the advent of novel ERG technologies, most of these barriers related to clinical ERG data acquisition have been removed (Nakamura et al., 2016; Asakawa et al., 2017; Kato et al., 2017; Hobby et al., 2018; Liu et al., 2018; Man et al., 2020), opening up the possibility to effectively use ERG data for glaucoma diagnostics, calling the necessity for the development of novel approaches (e.g., M-L-based ones) that is capable to quickly and thoroughly analyze such data.

Machine learning is based on statistical techniques to learn from data and develop predictive models (Jordan and Mitchell, 2015). Recently, there has been a surge of interest in machine learning as significant advancements in computational hardware (Shi et al., 2016) facilitate the development of novel machine learning approaches as solutions to problems in various disciplines from financial forecasting to public transportation and healthcare (Trafalis and Ince, 2000; Omrani, 2015; Ahmad et al., 2018). There are several predictive techniques in machine learning with various complexities, ranging from simple linear models to advanced non-linear models such as those based on deep learning algorithms (Shailaja et al., 2018; Khan et al., 2021; Saxe et al., 2021). Currently, available ERG analysis methods, such as those developed by Hood et al. (2000); Ventura and Porciatti (2006), have contributed to a significantly improved understanding of the relationship between ERG signals and vision loss. These methods are limited to frequency domain analysis (Miguel-Jiménez et al., 2010; Luo et al., 2011; Palmowski-Wolfe et al., 2011; Ledolter et al., 2013) and the analyses of differences in amplitude and latency of ERG (Fortune et al., 2002; Thienprasiddhi et al., 2003; Stiefelmeyer et al., 2004; Chu et al., 2007; Todorova and Palmowski-Wolfe, 2011; Ho et al., 2012; Hori et al., 2012). In addition, these methods are often time-consuming, labor-intensive, and focused on parameters developed to address a small subset of mostly genetic diseases of the eye affecting predominantly pediatric patient populations (Frishman et al., 2000; Graham et al., 2000; Dale et al., 2010). To achieve higher accuracy and a more detailed understanding of disease progression and of the impact of therapeutic intervention, more sophisticated features such as those obtained from wavelet analysis are required (Forte et al., 2008; Barraco et al., 2014). Additionally, currently available methods are often not suitable for analyzing large data sets and databases, rendering them incapable of taking advantage of complex and rich datasets (Consejo et al., 2019; Armstrong and Lorch, 2020). These drawbacks prompted others (Bowd et al., 2014; Yousefi et al., 2015; Atalay et al., 2016; Verma et al., 2017) and us to design and develop novel methods capable of handling complex and large datasets and ultimately to provide a unique approach for diagnosing early-stage glaucoma. However, it should be noted that early detection of glaucoma is not possible with currently available techniques during the early stages of glaucoma pathogenesis, when cellular changes occur that do not result in structural damage or visual impairment yet. Such early-onset factors predisposing to glaucoma development include processes preceding the onset of ocular hypertension, for example, the onset of iris pigment dispersion preceding IOP elevation in the DBA/2 mouse model. However, and more importantly, we identified cellular changes resulting in altered ERG signals, such as changes in oscillatory potentials, that currently cannot be detected with other functional or structural measures.

Boquete and colleagues developed a method to automate glaucoma diagnosis based on ERG signals using neural networks and structural pattern analysis (Boquete et al., 2012). They utilized thirteen features (morphological and transitional characteristics) for training the model and achieved a testing accuracy of 80.7% (Boquete et al., 2012). This study was limited to basic morphological characteristics of mfERG recordings (Boquete et al., 2012). Miguel-Jiménez et al. (2015) also employed neural networks for ERG-based glaucoma diagnosis but used continuous wavelet transformed coefficients and achieved a binary classification accuracy of 86.90% (Miguel-Jiménez et al., 2015). Although a higher accuracy was achieved, this analysis was limited to wavelet features only (Miguel-Jiménez et al., 2015). Nevertheless, both studies showed that machine learning-based methods trained even on compact data sets provide powerful tools to analyze ERG signals and provide potentially new information relevant for the early detection of glaucoma. Sarossy and colleagues investigated the relationship between a compact set of features and glaucoma that can be analyzed with machine learning approaches; however, the study was limited to the analysis of the photopic negative response (PhNR) and five additional features (Sarossy et al., 2021).

The goal of the present study was to comprehensively assess the capability of machine-learning-based methods to detect early-stage glaucoma using time-series ERG signals. In particular, the following points are addressed during method development:

1. Develop a framework to extract and identify important predictors (features) from ERG signals.
2. Compare the predictive capability of statistical and wavelet-based features for binary and multiclass classification.
3. Develop a robust ML-based model to diagnose glaucoma (binary classification).
4. Develop a robust ML-based model capable of distinguishing various stages of glaucoma progression (multiclass classification).
5. Develop a robust ML-based model to provide a quantitative assessment of visual function by predicting retinal ganglion cell count from ERG signals for the first time.

## 2. Methods

### 2.1. Overview

**Figure 1:**
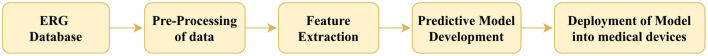
Machine learning workflow using ERG signals. *ERG Database:* the ERG database contains the input ERG data used to train the predictive model. *Pre-processing of data:* this step ensures data quality by transforming the data to a common baseline, accounting for missing data, and handling outliers. *Feature extraction:* mathematical operations are performed on the data to extract features/parameters that indicate functional deficits in the eye. *Predictive Model Development:* algorithms can determine trends and patterns in data from statistical analysis of extracted features during training; these models can predict either class or value from the input data are called classifier and regression models, respectively. *Deployment of Model into medical devices:* successful predictive models can be included with ERG testing devices to provide real-time prognosis and diagnosis.

ML based algorithms have been applied to Electrocardiogram (ECG) signals in order to develop predictive models for diagnosing heart diseases (Li et al., 2014; Al'Aref et al., 2019). Recently machine learning-based Artificial Neural Networks (ANN) have been applied to ERG signals for obesity diagnosis (Yapici et al., 2021). However, to date, machine learning-based methods have not been applied systematically to analyze ERG signals for glaucoma detection. Therefore, the potential of ERG signals in glaucoma diagnosis has not been fully utilized. The present work aims to develop a predictive model for early glaucoma diagnosis based on machine-learning algorithms by utilizing advanced features from ERG signals as predictors. The steps involved in developing a machine-learning-based predictive model for ERG analysis are shown in Figure 1. Each of these steps is explained in detail below.

### 2.2. ERG: A Biomarker

**Figure 2:**
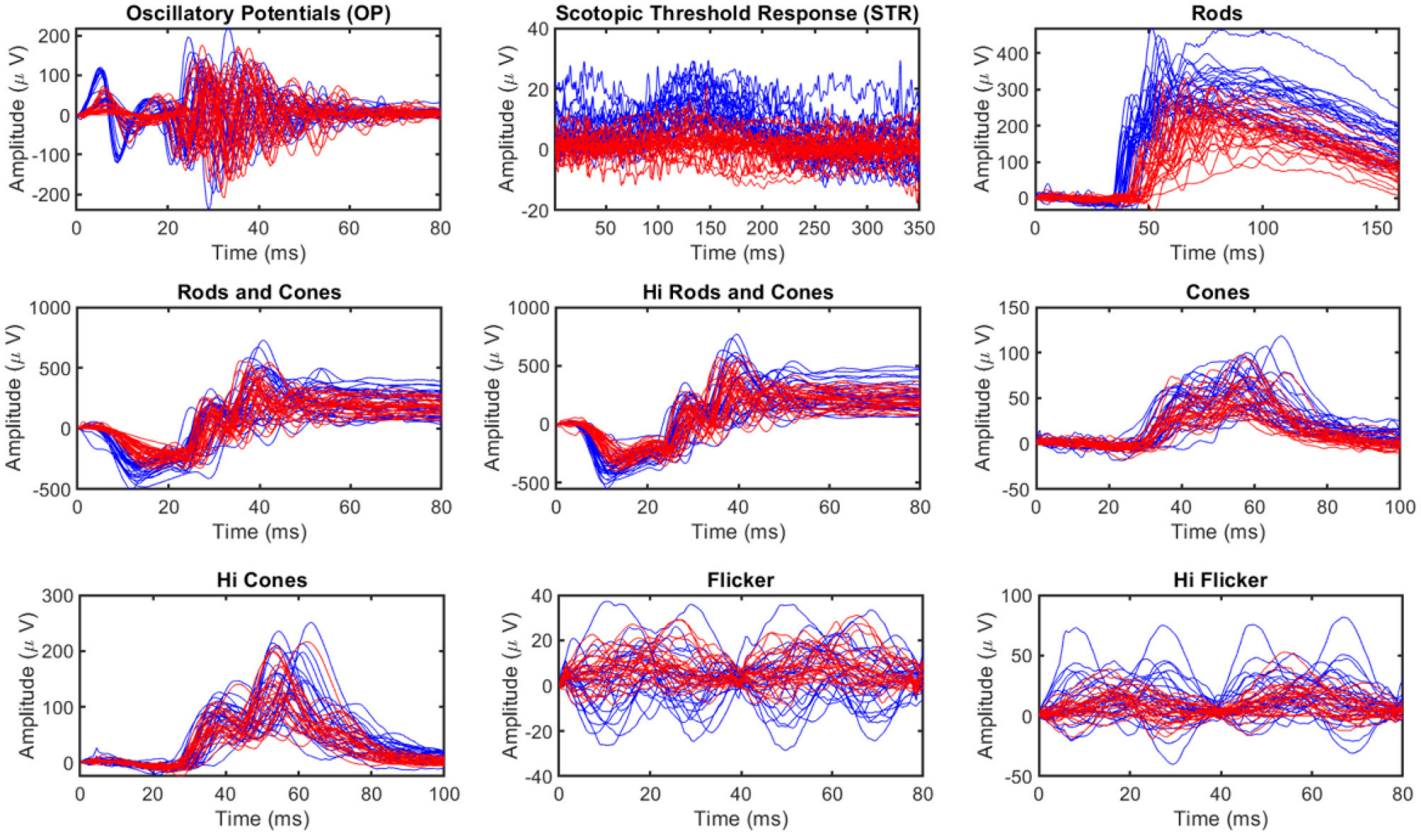
Visualization of ERG Signals manifesting their complex nature. The blue lines correspond to healthy and red lines correspond to glaucomatous Signals. Signals resulting from ERG tests include *OP* (Oscillatory Potential: small rhythmic wavelets superimposed on the ascending b-wave of the ERG), *STR* (Scotopic Threshold Response: negative corneal deflection elicited in the fully dark-adapted eye to dim stimuli), *Rods* (rod response), *Rods and cones* (standard rod-cone response), *Hi Rods and cones* (Hi-intensity rods and cones response), *Cones* (cone response), *Hi cones* (Hi-intensity cone response), *Flicker* (Flicker response), and *Hi flicker* (Hi-intensity flicker response).

Electroretinography measures the electrical responses of different types of cells in the retina, such as ganglion cells. These signals are usually measured in microvolts. Oscillatory Potential (OP) and Scotopic Threshold Response (STR) represent important ERG components indicative of RGC cell function (Saszik et al., 2002; Dong et al., 2004; Hancock and Kraft, 2004; Lei et al., 2006). OPs are small rhythmic wavelets superimposed on the ascending b-wave of the ERG and STR are negative corneal deflection elicited in the fully dark-adapted eye to dim stimuli. An International Society for Clinical Electrophysiology of Vision (ISCEV) standardized ERG protocol (Marmor et al., 2009) included several tests to measure the function of various retinal cell types, including the rod response, standard rod-cone response, Hi-intensity rods, and cones response, cone response, Hi-intensity cone response, flicker, and Hi flicker (Grillo et al., 2018). A visualization of nine ERG signals resulting from two ERG components (OP and STR) and seven ERG test responses is provided in Figure 2. The dynamics of ERG signals vary in people with various conditions and can therefore aid in differentiating individuals with glaucoma (Grillo et al., 2018), schizophrenia (Demmin et al., 2018), obesity (Yapici et al., 2021), and bipolar disorder (Hébert et al., 2020). ERG can also help in evaluating the effectiveness of new or existing drugs and therapy modalities (Lai et al., 2006, 2009; Nebbioso et al., 2009; da Silva et al., 2020).

### 2.3. Ganzfeld Flash Electroretinography

The development of pigmentary glaucomatous optic neuropathy in the DBA/2 mouse model had several similarities to glaucoma pathogenesis in human patients, including loss of vision and RGC (McKinnon et al., 2009; Burroughs et al., 2011; Grillo et al., 2013; de Lara et al., 2014; Kaja et al., 2014; Grillo and Koulen, 2015; Montgomery et al., 2016). The Ganzfeld flash electroretinography (fERG) procedures in mice were conducted under dim red light that was followed by an overnight dark adaptation (>12 h). Isoflurane at 3 and 1.5% was used respectively, to anesthetize mice and maintain anesthesia. The pupils were dilated using 1 drop of 1% tropicamide and were allowed to dilate for 10 min. Rectal temperature was monitored and maintained at 37°C using a heating pad. Silver-embedded thread electrodes were placed over the cornea in 1% methylcellulose with mini-contact lenses fitted preventing the corneal dehydration (Ocuscience LLC, Henderson, NV). The head was placed inside the Ganzfeld dome, and fERG with 2 recording channels was performed using an HMsERG system (Ocuscience LLC) equipped with an amplifier with a band pass from 0.3 to 300 Hz. Mice were subjected to the International Society for Clinical Electrophysiology of Vision (ISCEV) standardized ERG protocol [29], whose implementation is described in detail in Marmor et al. (2009). ERGView 4.380V software (OcuScience LLC) was used to perform statistical analyses including averaging multiple flashes recorded at each intensity and stored for further analysis. Additionally, mice were tested using a scotopic flash intensity series in the range of −4.5 to 1.5 log cd s/m^2^. Further, a 1:1,000 neutral density filter (ND3) was used to control the 7 lowest flash intensities; data were averaged from 10 flashes (−4.5 to −3.5 log cd s/m^2^), 4 flashes (−3 to 0.5 log cd s/m^2^) at the lower intensities or measured from 1 flash at the 2 highest intensities (1 to 1.5 log cd s/m^2^). Following the light adaptation (1.5 log cd s/m^2^ for 10 min), responses from a photopic series (−2 to 1.5 log cd s/m^2^; 32 flashes per intensity) were recorded in a separate fashion. Further details about data acquisition can be found in Grillo et al. (2018).

### 2.4. ERG Dataset

Ganzfeld fERG tests were performed on 4 months old (*n* = 15) and 11 months old (*n* = 15) male DBA/2 mice. Each animal had two sets of test data, one for each eye. Therefore, a total of 60 data sets for individual eyes were included in this study. Each data set comprised of nine different ERG signals (OP, STR, and seven signals from ERG testing protocols), as shown in Figure 2 (OPs are small rhythmic wavelets superimposed on the ascending b-wave of the ERG and STR are negative corneal deflection elicited in the fully dark-adapted eye to dim stimuli). Therefore, 540 recordings were utilized in this study. Intraocular pressure (IOP) and retinal ganglion cell (RGC) count measurements were also utilized in this study. Although IOP data was available for all animals, RGC counts were only available for 10 (20 eyes). The animals were grouped in a binary group (healthy and glaucomatous) based on age and multiclass group based on IOP as (normal, <12 mm Hg; high, [≥12 mm Hg <17 mm Hg]; glaucomatous, ≥17 mm Hg). All the data used in this study was well-balanced for respective groups.

### 2.5. Pre-processing of Data

ERG raw data may contain several anomalies such as different start times, missing data, different sampling frequencies, noise, and unequal lengths of the signal recordings. In Machine learning-based modeling, the quality of the training data can significantly impact the model performance. Therefore, pre-processing (data preparation and screening) is crucial to ensure the quality of the training dataset (Jambukia et al., 2015). Pre-processing steps considered in the present study include,

1. Baseline adjustment
2. Feature extraction
3. Handling missing data
4. Handling outliers
5. Feature scaling
6. Feature selection

The signal's baseline (start time) can be different for different animals and testing protocols. Therefore, all the measurements were brought to a common baseline (start time was offset to zero) during baseline adjustment (Jambukia et al., 2015). Feature extraction involves computing a reduced set of values from a high-dimensional signal capable of summarizing most of the information contained in the signal (Khalid et al., 2014). The missing data were replaced with mean values (Graham et al., 2013). For handing outliers, values more than three scaled median absolute deviations (MAD) away from the median were detected as outliers and replaced with threshold values used in outlier detection (Aguinis et al., 2013). The feature's values vary widely, even by orders of magnitude. Therefore, it is important to bring the feature values to a similar range (feature scaling), especially when using distance-based machine learning algorithms (Wan, 2019). Feature selection is further dimensionality reduction from the extracted features. It is performed to reduce the computational cost of modeling, to achieve a better generalized, high-performance model that is simple and easy to understand (Aha and Bankert, 1996). Feature extraction and selection are explained in detail in the following sections.

### 2.6. Feature Extraction

**Figure 3:**
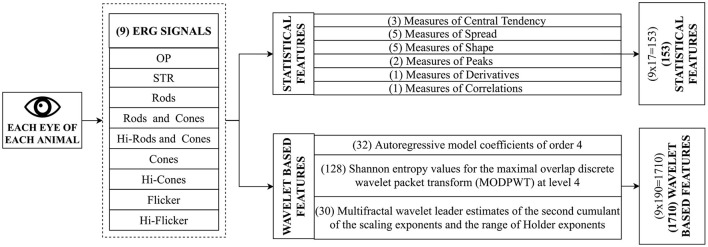
Feature Extraction. During this process, mathematical operations are performed on the data to extract features. This step is crucial for discovering features indicative of functional deficits in the eye. ERG test on each eye leads to nine signals, as shown in Figure 2. Two sets of features (Standard features and advanced features) are extracted from each of the nine signals. The standard set of features include common statistical features such as mean, quartiles, and entropies. In contrast, the advanced set of features include sophisticated features such as autoregressive coefficients, Shannon entropy, and wavelet features.

ERG signals are complex high-dimensional data, and training a model with many variables requires significant computational resources. Feature extraction reduces the dimensionality of the data by computing a reduced set of values from a high-dimensional signal capable of summarizing most of the information contained in the signal (Guyon et al., 2008). In the present study, feature extraction was performed in two phases. First, common statistical features were extracted from the signal, followed by the extraction of advanced wavelet-based features. Figure 3 provides an overview of the feature extraction process and is explained below.

#### 2.6.1. Statistical Feature Extraction

A total of 17 Statistical features capable of describing the general behavior of ERG signals were extracted from the signal. These features were grouped as follows.

1. Measures of Central Tendency
2. Measures of Spread
3. Measures of Shape
4. Measures of Peaks
5. Measures of Derivatives
6. Measures of Correlation

Measures of central tendency included mean, median, trimmed mean. Measures of spread included range, standard deviation, variance, mean absolute deviation, and interquartile range. Measures of the shape include skewness, kurtosis, central moments of the second and third-order, and aspect ratio. Measures of peaks included the number of peaks and troughs in the signal. Measures of derivatives include the first-order derivative of the signal with respect to time. Measures of correlation included the correlation coefficient of the signal with respect to time. The equations for the computation of these quantities can be found in Asgharzadeh-Bonab et al. (2020); Yapici et al. (2021).

#### 2.6.2. Advanced Feature Extraction

Advanced features capable of capturing subtle changes were extracted from the signal. Each signal was split into 32 blocks (~ 2000 samples/block) to further capture subtle changes in the signal (Martis et al., 2014). Daubechies least-asymmetric wavelet with four vanishing moments (Symlets 4) was used as mother wavelet to derive the wavelet coefficients (Daubechies, 1992). The following features (190 features in total as shown in Figure 3) were extracted from each block of the signal:

**AR coefficients:** The signal *x*[*n*] at time instant n in an AR process of order p can be described as a linear combination of *p* earlier values of the same signal. The procedure is modeled as follows:


(1) $$\begin{array}{l} x\left[n\right]=\overset{p}{\underset{i=1}{\sum }}a\left[i\right]x\left[n-i\right]+e\left[n\right] \end{array}$$

where *a*[*i*] is the AR model's *i*^*th*^ coefficient, *e*[*n*] denotes white noise with mean zero, and *p* denotes the AR order. The AR coefficients for each block were estimated using the Burg method (Zhao and Zhang, 2005); the order was determined using the ARfit model order selection method (Neumaier and Schneider, 2001) as 4th order. Therefore a 4-order AR model is chosen to represent each of the ERG signal components.

**Wavelet based Shannon Entropy:** The Shannon entropy is an information-theoretic measure of a signal. Shannon entropy (denoted as SE) values for the maximal overlap discrete wavelet packet transform (MOD- PWT) using four-level wavelet decomposition was computed on the terminal nodes of the wavelet (Li and Zhou, 2016). Mathematical expression for Shannon entropy using wavelet packet transform is as follows:


(2) $$\begin{array}{l} SE_{j}=-\overset{N}{\underset{k=1}{\sum }}p_{j,k}*\log p_{j,k} \end{array}$$

where *N* is the number of coefficients in the *j*^*th*^ node and *p*_*j,k*_ are the normalized squares of the wavelet packet coefficients in the *j*^*th*^ terminal node of the wavelet.

**Multifractal wavelet leader estimates and multiscale wavelet variance estimates:** The multifractal measure of the ERG signal was obtained using two wavelet methods (wavelet leader and cumulant of the scaling exponents). Wavelet leaders are time/space-localized suprema of the discrete wavelet coefficients' absolute value. These suprema are used to calculate the Holder exponents, which characterize the local regularity. In addition, second cumulant of the scaling exponents were obtained. Scaling exponents are scale-dependent exponents that describe the signal's power-law behavior at various resolutions. The second cumulant basically depicts the scaling exponents' divergence from linearity (Leonarduzzi et al., 2010). Wavelet variance of ERG signals were also obtained as features. Wavelet variance quantifies the degree of variability in a signal by scale, or more precisely, the degree of variability in a signal between octave-band frequency intervals (Maharaj and Alonso, 2014).

### 2.7. Feature Selection

Feature extraction discussed previously was performed in order to reduce the dimensionality of the signals; however, the resulting number of features was still higher than the number of training data. Therefore, further reduction in the dimensionality of the data was performed using the feature selection method to identify relevant features for classification and regression. It should be noted that feature selection was necessary to reduce the computational cost of modeling, prevent the generation of a complex and over-fitted model with high generalization error, and generate a high-performance model that is simple and easy to understand (Saeys et al., 2007). In particular, the Minimum Redundancy Maximum Relevance (MRMR) sequential feature selection algorithm was used in the present study because this algorithm is specifically designed to drop redundant features [see (Darbellay and Vajda, 1999; Ding and Peng, 2005) for mathematical details/formulations], which was required to design a compact and efficient machine-learning-based model (Zhao et al., 2019). It is worth noting that other available dimensionality reduction techniques such as Principal component analysis (PCA) were not considered in this study as such techniques do not allow for direct tracing and understanding the relevance of each feature (Aha and Bankert, 1996).

### 2.8. Predictive Model Development

ML models are mathematical algorithms that provide predictions based on an inference derived from the generalizable predictive patterns of the training data (Bzdok et al., 2018). Several machine learning models were employed and evaluated in order to identify the best one to classify the ERG signals. These included decision trees, discriminant, support vector machine, nearest neighbor, and ensemble classifiers. Most of these models can perform both classification and regression. Decision tree-based models predict the target variable by learning simple decision rules (Navada et al., 2011). Discriminant classifiers are based on the assumption that each class has different Gaussian distributions of data, and the classification is performed based on Gaussian distribution parameters estimated by the fitting function (Cawley and Talbot, 2003). Support vector machine (SVM) is based on Vapnik–Chervonenkis theory, where a hyperplane separating the classes is determined. SVMs are efficient algorithms suitable for compact datasets (Noble, 2006). The nearest neighbor algorithm is based on the assumption that similar things exist nearby. It is a simple yet versatile model with high computational cost (Zhang and Zhou, 2007). Ensemble methods such as bagged trees (or random forest) combine the predictions of several learning algorithms with improving generalization. Although these methods are also computationally expensive, they are unlikely to over-fit (Dietterich, 2000). Regression analysis based on the above techniques was also performed alongside classification.

### 2.9. Performance Evaluation

Various performance evaluation metrics were utilized to compare different machine learning algorithms. The metrics used in this study include accuracy, sensitivity, specificity, precision, recall, f-score, root mean squared error, and their corresponding mathematical formulations are given below.

The abbreviations used in the following expressions include True Positive (TP) which are the cases the model correctly predicted the positive (glaucomatous) class. True Negative (TN) are the cases the model correctly predicted the negative (non-glaucomatous) class. False Positive (FP) are the cases the model incorrectly predicted the positive (glaucomatous) class. False Negative (FN) are the cases the model incorrectly predicted the negative (non-glaucomatous) class.

#### 2.9.1. Accuracy

Accuracy is the percentage of correctly classified observations, as shown below.


(3) $$\begin{array}{l} \text{Accuracy}\left(\%\right)=\frac{\text{TP }+\text{ FP}}{\text{TP }+\text{ TN }+\text{ FP }+\text{ FN}} \end{array}$$

#### 2.9.2. Sensitivity

Sensitivity/Recall estimates the proportion of actual positives (e.g., actual glaucomatous) was identified correctly.


(4) $$\begin{array}{l} \text{Sensitivity}/\text{Recall }\left(\text{RE}\right)=\frac{\text{TP}}{\text{TP }+\text{ FN}} \end{array}$$

#### 2.9.3. Specificity

Recall estimates the model's ability to correctly reject healthy patients without a Glaucoma.

#### 2.9.4. Precision

Precision estimates the proportion of positive predictions (e.g., glaucomatous predictions) that was actually correct.


(5) $$\begin{array}{l} \text{Precision }\left(\text{PR}\right)=\frac{\text{TP}}{\text{TP }+\text{ FP}} \end{array}$$

#### 2.9.5. F-Score

The F-Score estimates the harmonic mean of the precision and recall.


(6) $$\begin{array}{l} \text{F- Score}=\frac{\text{PR }\times \text{ RE}}{\text{PR }+\text{ RE}} \end{array}$$

#### 2.9.6. Root Mean Square Error (RMSE)

The Root Mean Square Error (RMSE) was used as the performance evaluation metric for regression analysis. RSME is the standard deviation of the prediction errors (residuals).


(7) $$\begin{array}{l} \text{RMSE}=\sqrt{\frac{\sum_{i=1}^{N}{\left(\text{Actual }x_{i}-\text{Predicted }{\hat{x}}_{i}\right)}^{2}}{N}} \end{array}$$

Where *N* is the number of observations.

## 3. Results

A machine learning-based approach was developed and trained using the balanced ERG data previously published by Grillo et al. (2018). Although a compact dataset of 60 observations and 540 signals was used in this study, the current framework was able to consistently detect features (Figures 6, 9) that are known to be medically relevant such as OP, STR, Flicker reported in various studies (Tyler, 1981; Saszik et al., 2002; de Lara et al., 2014, 2015; Porciatti, 2015; Grillo et al., 2018). In particular, studies conducted by Wilsey and Fortune (2016); Hermas (2019); Beykin et al. (2021) investigating the variability of PhNR in glaucomatous and healthy subjects in PERG and fERG have found that PhNR to be an important biomarkers for detection of glaucoma. It is worth noting that in fERG analysis (ERG protocol for this study), pSTR, nSTR, PhNR are extracted from STR.

Therefore, we were able to demonstrate that the proposed framework for early-stage glaucoma diagnosis can be reproducibly evaluated and validated even on such a compact database. Furthermore, we would like to note that there are other investigations that successfully applied ML-based method in different fields, including biomedical (Seo et al., 2020) and material science (Zhang and Ling, 2018) using compact datasets. The procedure employed for the development of the predictive modeling framework is summarized below.

- **Data Split:** Hold out (80% training, k-fold cross-validation, 20% testing).
- **Dimensionality reduction:** Feature Extraction.
- **Feature selection:** MRMR.
- **Hyper-parameter tuning:** k-fold cross-validation (*k* = 10).
- **Model Evaluation:** Performance metrics evaluated on the unseen testing set.

The dataset was divided into two parts; 80% of the data was used for training and validation, and the remaining 20% was set aside for testing. The hold-out testing strategy ensured that the test data was never a part of the training process (Yadav and Shukla, 2016). Dimensionality reduction was performed using feature extraction and feature selection. MRMR feature selection algorithm was used to identify the important predictors. K-fold (*K* = 10) cross-validation was used for training and hyper-parameter tuning (Duan et al., 2003). The cross-validation technique significantly reduces bias when working with small datasets (Varma and Simon, 2006). The loss function was the objective minimization function for both classification regressions during hyper-parameter optimization. The hyper-parameters associated with corresponding ML algorithms (Feurer and Hutter, 2019), as shown in Table 1, were optimized through nested cross-validation. Next, the trained model with optimized hyper-parameters was evaluated using test data that was not a part of training. To further ensure that the machine learning models compared in this investigation were not over-fitted, given the compact dataset used in the present study, the behavior of training and testing error vs. training cycles was monitored. Different techniques, including Tree, Discriminant, SVM, Naive Bayes, Tree Ensemble, and KNN, were applied, and their performances were assessed. The performance of each technique was assessed based on the accuracy (discussed in section 2.9) is tabulated in Table 2. Considering binary and multiclass classifications, it can be seen that the Ensemble-based technique (bagged tree) was consistently outperforming other techniques. Additionally, other performance metrics for ensemble bagged trees (discussed in section 2.9) are summarized in Table 3.

**Table 1:** Hyperparameters tested/optimized.

**Method**	**Hyperparameter search range**	**Optimized hyperparameters**
Ensemble	**Ensemble method:** Bag, GentleBoost, LogitBoost, AdaBoost, RUSBoost **Number of learners:** 10–500 **Learning rate:** 0.001-1 **Maximum number of splits:** 1–47 **Number of predictors to sample:** 1–5	**Ensemble method:** Bag **Maximum number of splits:** 1 **Number of learners:** 52 **Number of predictors-** **to sample:** 1
Knn	**Number of neighbors:** 1-24 **Distance metric:** City block, Chebyshev, Correlation, Cosine, Euclidean, Hamming, Jaccard, Mahalanobis, Minkowski (cubic), Spearman **Distance weight:** Equal, Inverse, Squared inverse **Standardize data:** true, false	**Number of neighbors:** 24 **Distance metric:** Correlation **Distance weight:** Inverse **Standardize data:** true
NaiveBayes	**Distribution names:** Gaussian, Kernel **Kernel type:** Gaussian, Box, Epanechnikov, Triangle	**Distribution names:** Gaussian **Kernel type:** Epanechnikov
Discriminant	**Discriminant type:** Linear, Quadratic, Diagonal Linear, Diagonal Quadratic	**Discriminant type:** Diagonal Linear
SVM	**Multiclass method:** One-vs-All, One-vs.-One **Box constraint level:** 0.001-1000 **Kernel scale:** 0.001–1000 **Kernel function:** Gaussian, Linear, Quadratic, **Cubic Standardize data:** true, false	**Kernel function:** Linear **Box constraint level:** 2.4185 **Multiclass method:** One-vs.-All **Standardize data:** false
Tree	**Maximum number of splits:** 1–47 **Split criterion:** Gini's diversity index, Maximum deviance reduction	**Maximum number of splits:** 5 **Split criterion:** Maximum deviance reduction

**Table 2:** Testing accuracy obtained using various machine learning techniques.

		**Tree**	**Discriminant**	**SVM**	**Naive Bayes**	**Ensemble (Bagged)**	**KNN**
**Binary**	**Statistical**	75	80	**83.33**	80	**83.33**	66.70
	**Wavelet**	83.33	83.33	**91.70**	83.33	**91.70**	75
**Multiclass**	**Statistical**	33.33	41.70	50	16.70	**53.33**	33.33
	**Wavelet**	41.70	50	64.66	33.33	**80**	50

*Values in bold font indicate the accuracies of best-performing classifier*.

**Table 3:** Performance metrics for ensemble classifier.

		**Accuracy**	**F-measure**	**Precision**	**Sensitivity**	**Specificity**
**Binary**	**Statistical**	80	80	80.36	80.36	80.36
	**Wavelet**	91.67	91.61	92.86	91.67	91.67
**Multi-class**	**Statistical**	53.33	50.74	53.18	51.67	75.48
	**Wavelet**	80	79.63	83.81	83.333	90.30

### 3.1. Binary Classification

**Figure 4:**
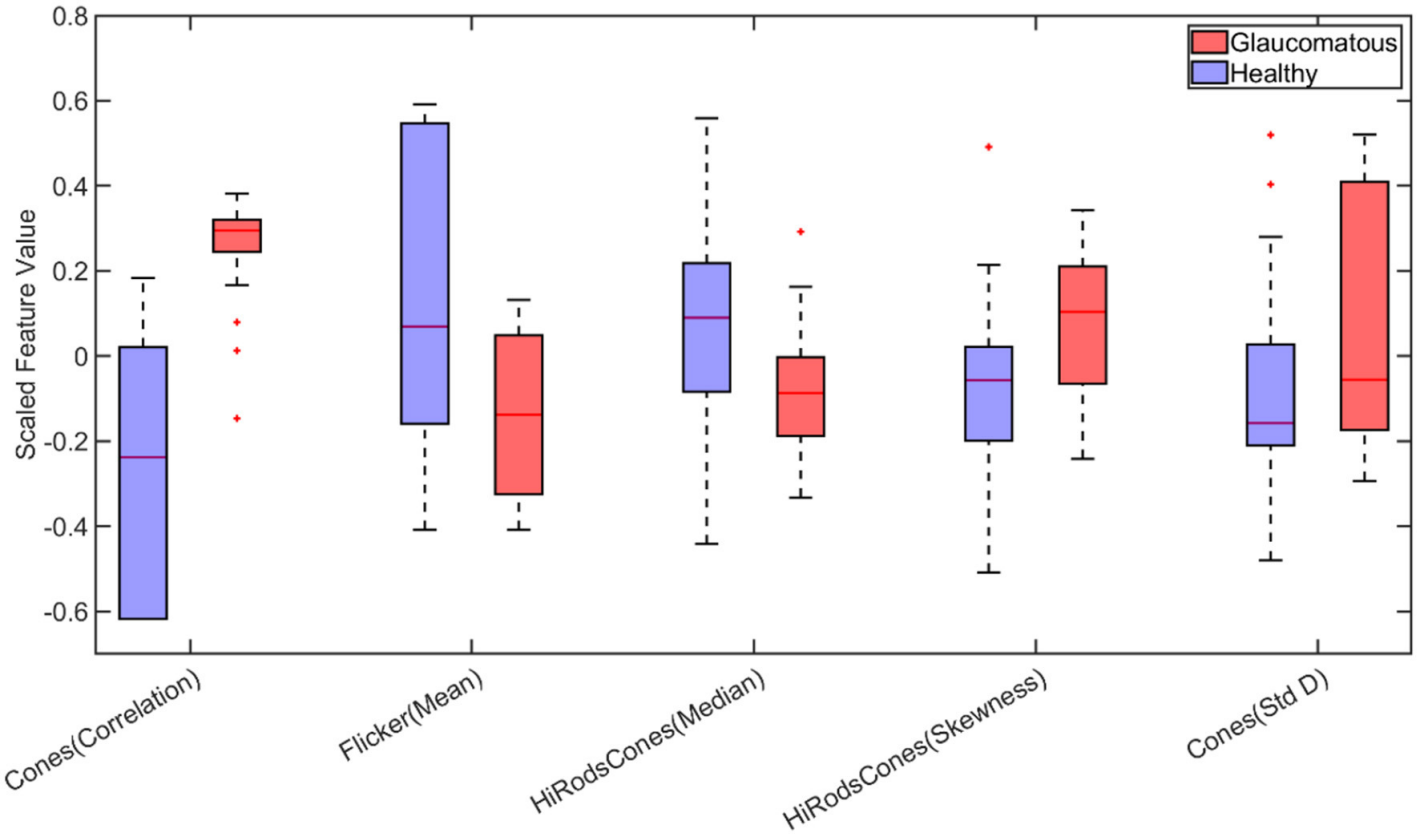
Boxplot of statistical features selected by Minimum Redundancy and Maximum Relevance (MRMR) feature selection algorithm for binary classification (Std D, Standard Deviation). On each box, the central mark indicates the median, and the bottom and top edges of the box indicate the 25th and 75th percentiles, respectively. The whiskers extend to the most extreme data points not considered outliers, and the outliers are plotted individually using the “+” marker symbol.

For binary classification (classifying animals with/without glaucoma) based on statistical features, the correlation of cones, mean of flicker, median, and skewness of Hi Rods and cones, and standard deviation of cones were identified as important among the statistical features as shown in Figure 4. Moreover, the box plot demonstrates variations of each feature for each class (with/without glaucoma), respectively. Several models, including SVM and ensemble-based classifiers were used for training, and their performances were assessed. It turned out that the SVM and ensemble bagged tree provide the best performance with a testing accuracy of 83.33%, as shown in Table 2.

**Figure 5:**
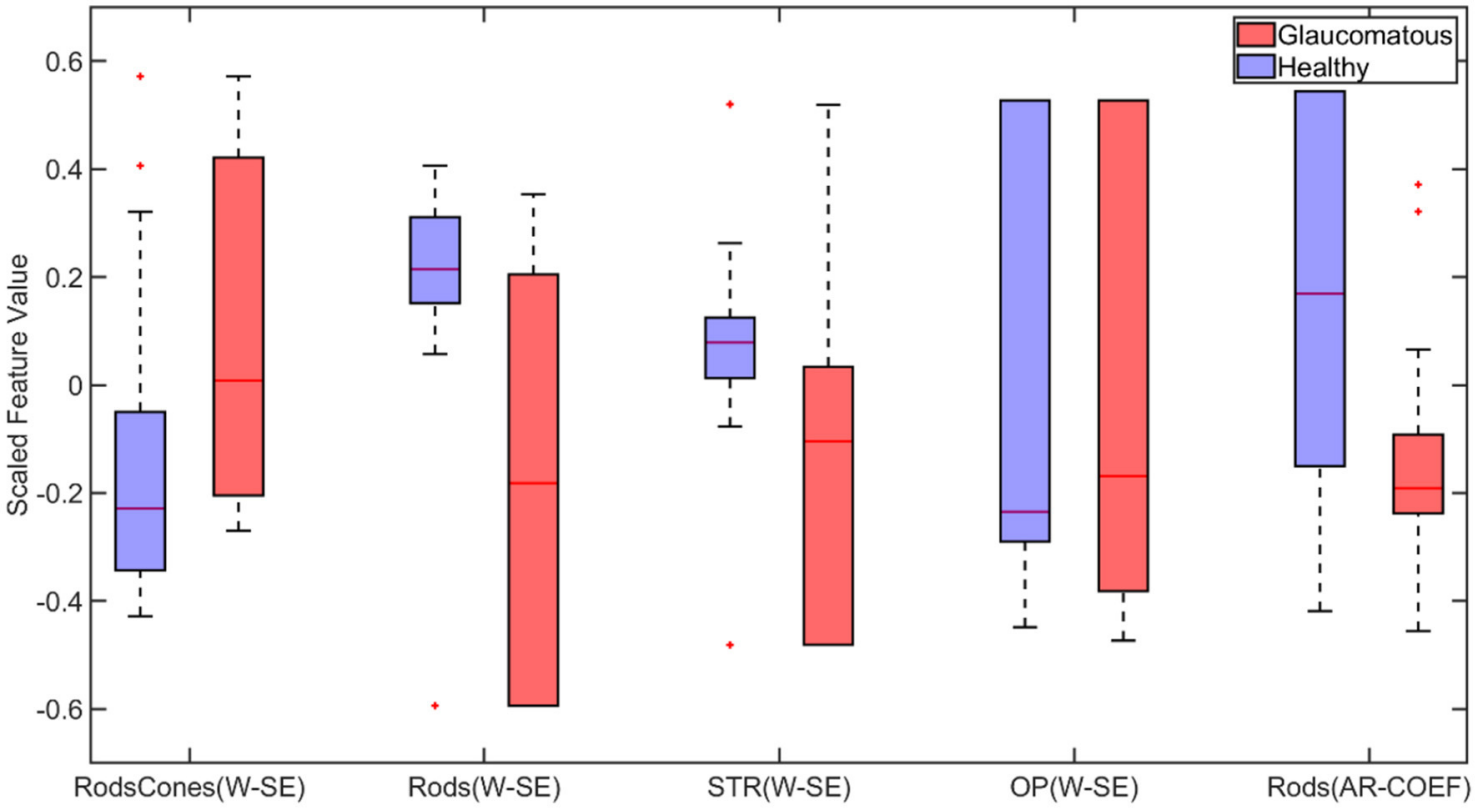
Box plot of wavelet-based features selected by Minimum Redundancy and Maximum Relevance (MRMR) feature selection algorithm for binary classification (W-SE, Wavelet based Shannon Entropy; AR-COEF, Autoregressive Coefficient). On each box, the central mark indicates the median, and the bottom and top edges of the box indicate the 25th and 75th percentiles, respectively. The whiskers extend to the most extreme data points not considered outliers, and the outliers are plotted individually using the “+” marker symbol.

Next, the binary classification was performed using wavelet-based features. Among the extracted wavelet features, Shannon Entropy Values for Maximal Overlap Discrete Wavelet Packet Transform (MOD-PWT) were identified as important features from Rods and cones, Rods, STR, and OP, as shown in Figure 5. The utilization of the selected advanced features improved the accuracy to 91.70% by the ensemble bagged tree algorithm.

**Figure 6:**
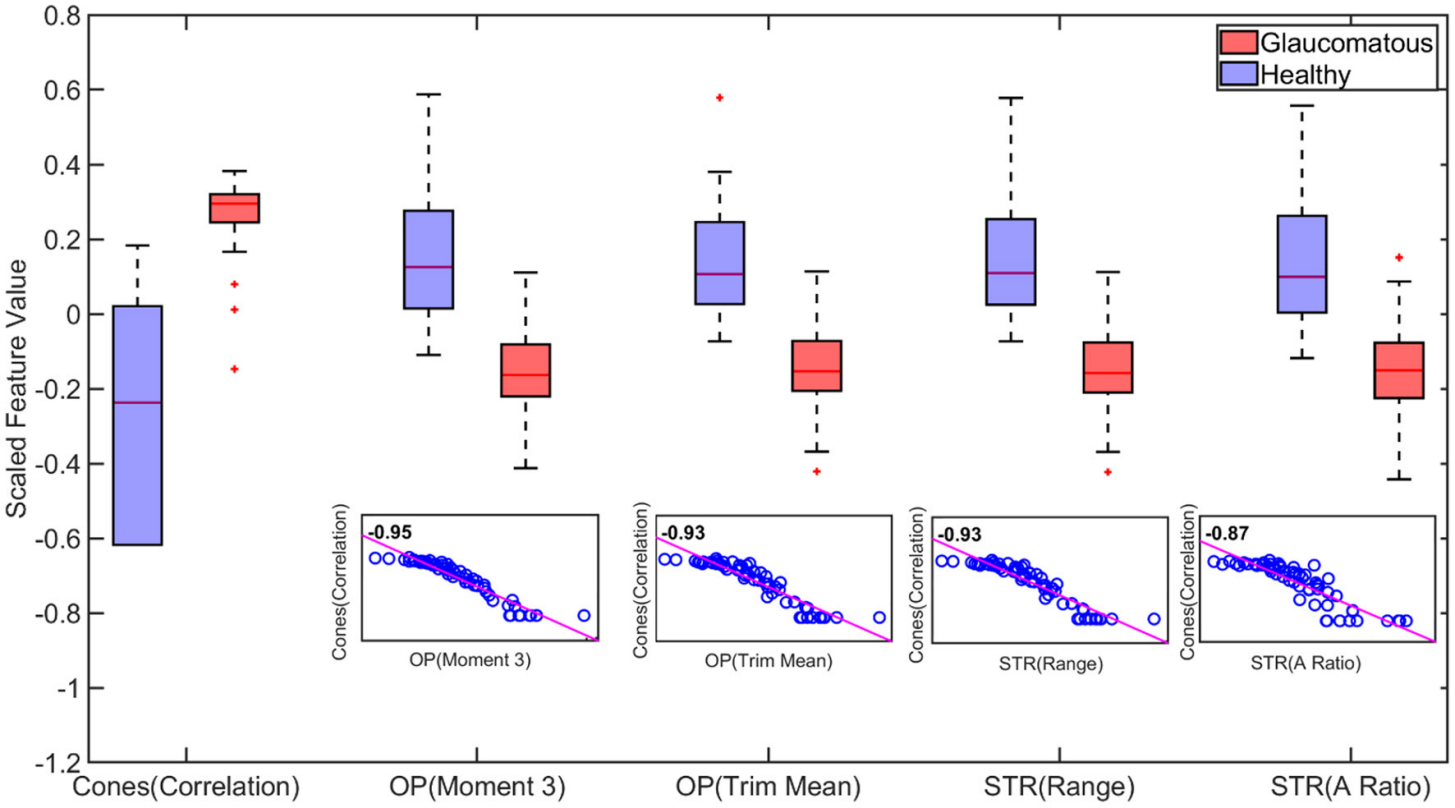
Boxplot of statistically important features for binary classification. The important features capable of distinguishing healthy and glaucomatous are correlated feature from Cones, third order Moment and trimmed mean feature from Oscillatory Potentials (OP) and Range and aspect ratio from Scotopic Threshold Response (STR). However, the high similarity between these features quantified by the correlation scores in the scatter plot create redundancy (inclusion cones(correlation) feature alone vs inclusion all five features does not improve accuracy). Therefore, utilizing the cones correlation feature alone captures the behavior of the other four features. This dropping of redundant features and choosing Cones (correlation) feature alone is achieved by using Minimum Redundancy and Maximum Relevance (MRMR) algorithm (On each box, the central mark indicates the median, and the bottom and top edges of the box indicate the 25th and 75th percentiles, respectively. The whiskers extend to the most extreme data points not considered outliers, and the outliers are plotted individually using the “+” marker symbol.).

It should be noted that the MRMR method selects features based on statistical relevance while dropping redundant features and thus, is computationally efficient (Darbellay and Vajda, 1999; Ding and Peng, 2005). Figure 6 demonstrates this for binary classification. It can be observed that correlation feature from cones, Moment of order three and trimmed mean feature from Oscillatory Potentials (OP) and Range and aspect ratio from Scotopic Threshold Response (STR) are highly correlated; Therefore, only the feature cones correlation was picked by the MRMR algorithm as inclusion of the other three did not increase/decrease the models predictability.

**Figure 7:**
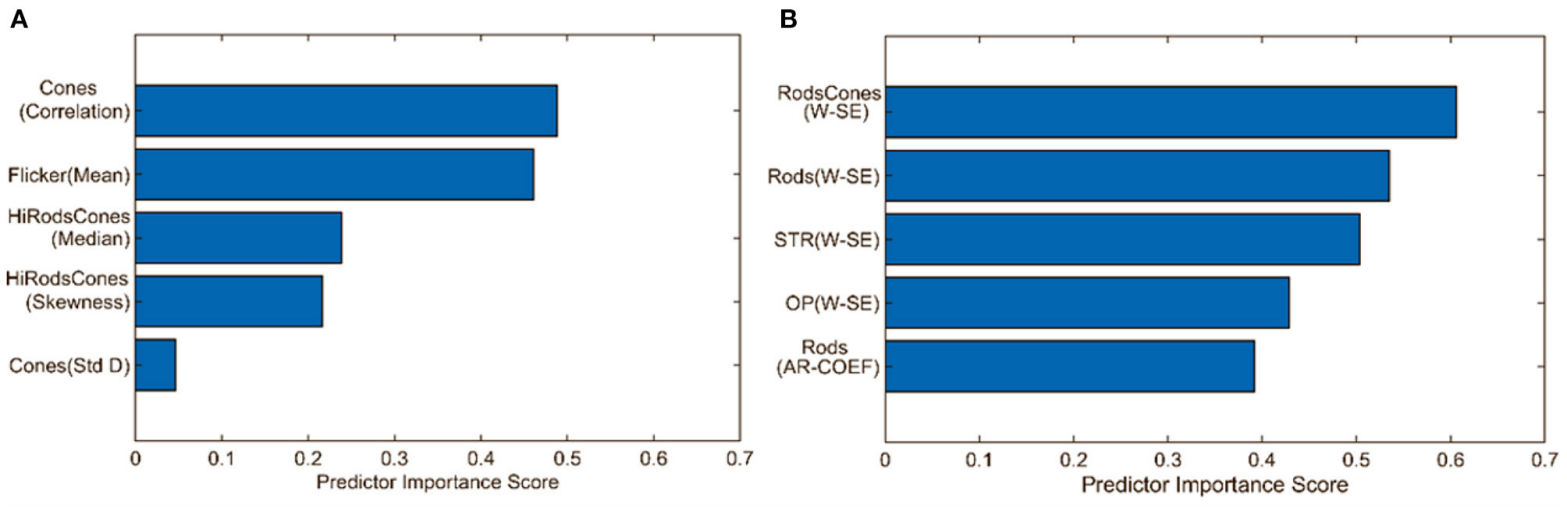
Comparison of predictive importance scores for binary classification using **(A)** statistical features and **(B)** wavelet-based features. This bar chart illustrates the superior predictive capability of wavelet-based features. Std D, Standard Deviation; W-SE, Wavelet based Shannon entropy; AR-COEF, Autoregressive coefficient.

Figure 7 compares the predictive importance scores obtained based on the statistical and wavelet-based features. Predictive importance scores describe the predictive capability of selected features (Kuhn and Johnson, 2013). It can be observed that wavelet-based features can distinguish healthy and glaucomatous animals suggesting that they are more sensitive to subtle changes in ERG signals due to glaucoma. It should be noted that the feature selection algorithm MRMR (Maximum Relevance and Minimum Redundancy) ignores highly correlated features for model simplicity. Therefore, only uncorrelated sets of features that improved predictability across the animals were chosen, i.e., for a set of correlated features, one representing the correlated set gets picked by the algorithm. Figure 6 demonstrates the list of important but highly correlated features that were dropped. The scatter plot inside the Figure 6 shows the correlation coefficients confirming the high degree of the correlation between them.

### 3.2. Multiclass Classification

**Figure 8:**
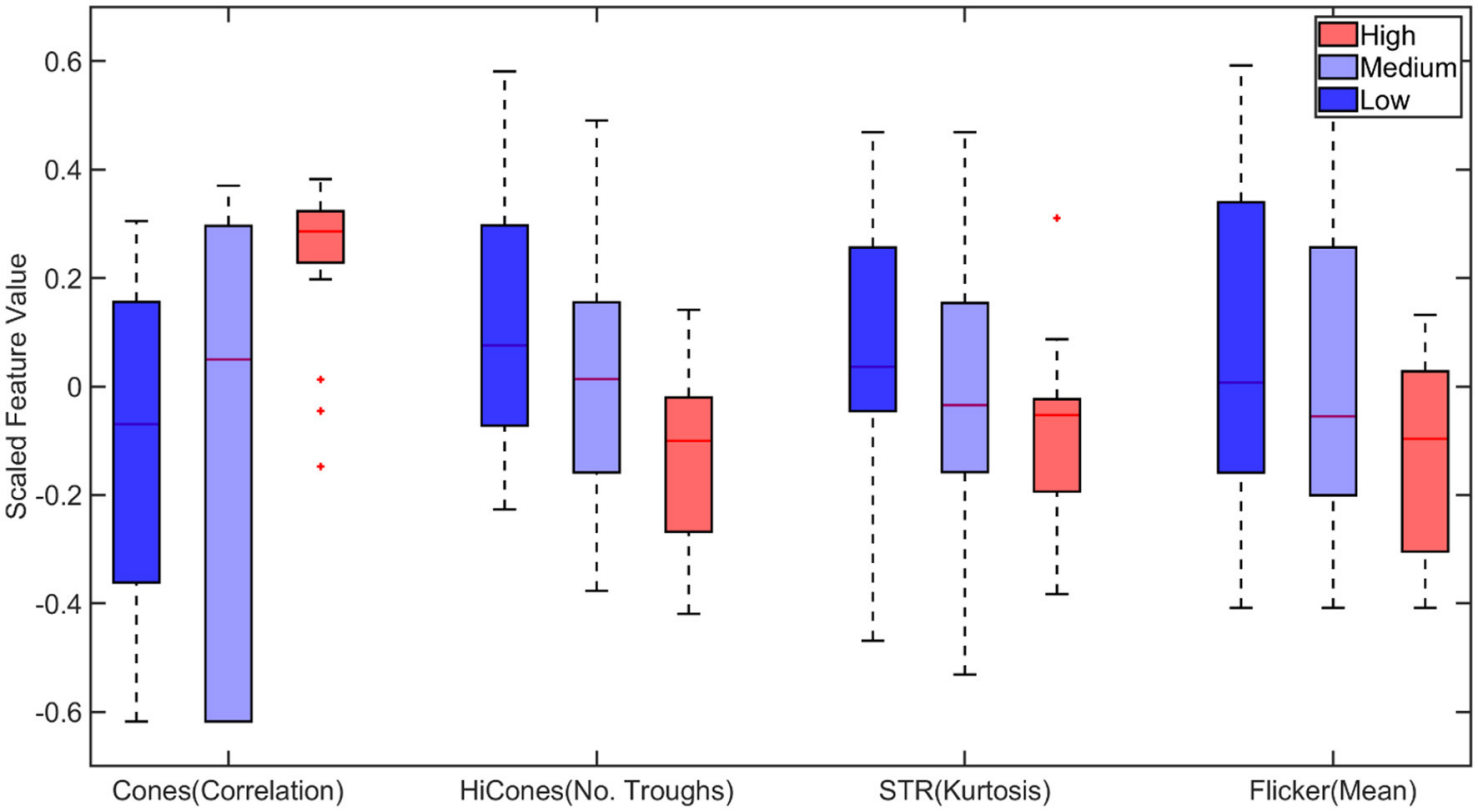
Boxplot of statistical features selected by Minimum Redundancy and Maximum Relevance (MRMR) feature selection algorithm for multiclass classification. STR, Scotopic Threshold Response. On each box, the central mark corresponds to the median, and the bottom and top edges of the box correspond to the 25th and 75th percentiles, respectively. The dashed lines (whiskers) extend to the most extreme data points not considered outliers, and the outliers are plotted individually using the “+” marker symbol.

For multiclass classification (classifying animals to different stages, normal, high, and glaucomatous as mentioned in section 2.4) based on statistical features, the correlation of cones, number of troughs in Hi cones, kurtosis of STR and mean of flicker were identified as important among the statistical features as shown in Figure 8. Several models, including SVM and ensemble-based classifiers, were used for training, and their performances were assessed. It turned out that the ensemble-based classifiers, specifically the bagged trees model, provided the best performance with a testing accuracy of 53.33%, as shown in Table 2.

**Figure 9:**
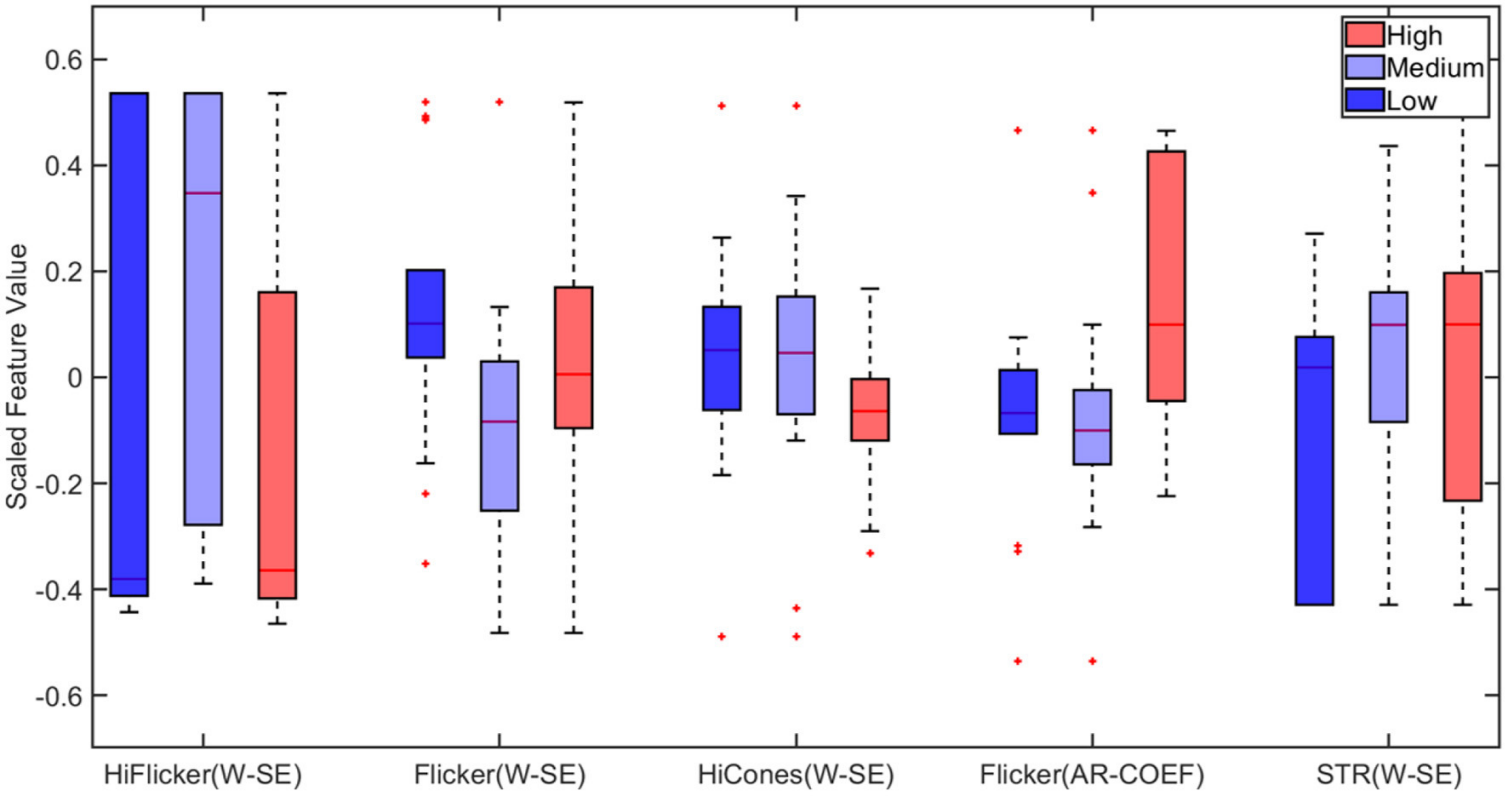
Boxplot of wavelet-based features selected by Minimum Redundancy and Maximum Relevance (MRMR) feature selection algorithm for multiclass classification. STR, Scotopic Threshold Response; W-SE, Wavelet based Shannon Entropy; AR-COEF, Autoregressive Coefficient. On each box, the central mark indicates the median, and the bottom and top edges of the box indicate the 25th and 75th percentiles, respectively. The whiskers extend to the most extreme data points not considered outliers, and the outliers are plotted individually using the “+” marker symbol.

Next, the multiclass classification was performed using wavelet-based features. Among the extracted wavelet features, Wavelet variance of rods and Shannon Entropy Values and AR coefficients for Maximal Overlap Discrete Wavelet Packet Transform (MOD-PWT) were identified as important features from Hi-Flicker, Flicker, Hi-cones, and STR as shown in Figure 9. The identification of flicker as an important distinguishing feature in diagnosing early-stage glaucoma was consistent with previous studies (Tyler, 1981; Lachenmayr and Drance, 1992; Horn et al., 1997; Yoshiyama and Johnson, 1997). In fact, flicker measurements in eyes with early-stage glaucoma exhibited a loss in sensitivity around 30–40 Hz (Tyler, 1981). It is worth noting that the flicker measurements used in this study were recorded using flashes at 30 Hz. The identification of the flicker ERG test and the corresponding features, among other tests, reconfirmed the capability of the current approach in identifying the relevant features. Training the ensemble bagged trees model, utilizing the selected advanced features, improved the multiclass classification accuracy to 80%, as shown in Table 2. This improvement in accuracy indicated that wavelet-based features can distinguish healthy and glaucomatous animals suggesting that they are more sensitive to subtle changes in ERG signals due to glaucoma. The multiclass classification ability of this framework reaffirmed the rich and complex nature of ERG signals in assessing the disease progression.

### 3.3. RGC Regression

**Figure 10:**
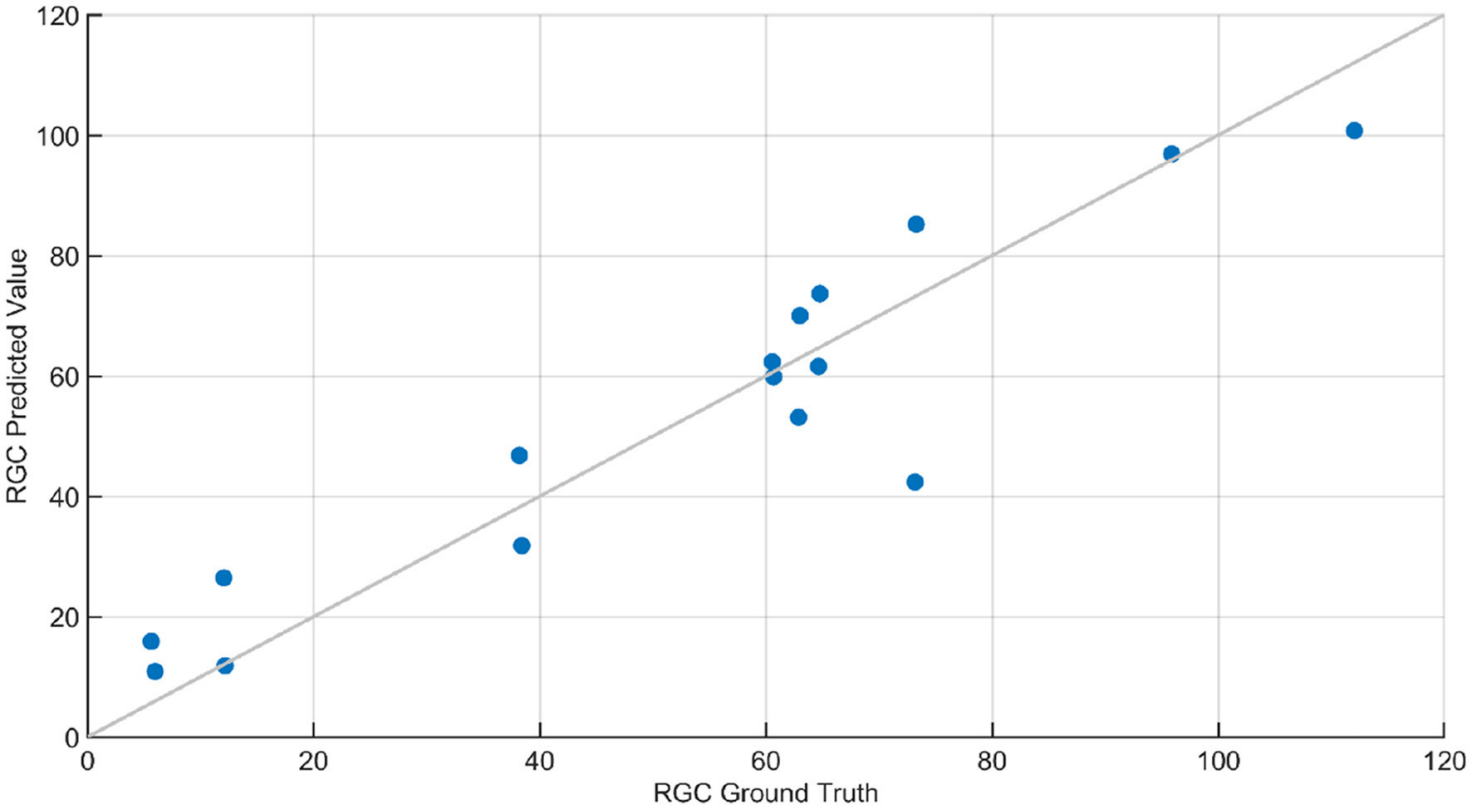
RGC count regression plot. This plot contains the ground truth and predicted response of RGC count predicted using Gaussian Process Regression (GPR). The squared exponential GPR model was trained using both standard and advanced features. The RGC count of the animals ranged between 8 and 120, and the root mean squared error in the prediction of RGC was 11.2. The line in this plot denotes when the predicted values are equal to ground truth values.

Regression analysis was performed to predict retinal ganglion cell count from ERG signals. Feature selection for regression was performed using MRMR sequential feature selection. RGC values of the animals ranged between 8 and 120. RSME for RGC regression was 15.64 and 11.20 for models trained with statistical features and wavelet-based features, respectively. Regression results using wavelet-based features are shown in Figure 10. The results in Grillo et al. (2018) indicate that RGC counts had a strong correlation with STR and OPs. The dominant features selected for RGC regression (from STR and OP) were in agreement with the findings in Grillo et al. (2018). Table 4 compares performance of various ML based regression models in predicting retinal ganglion cells (RGCs) counts: The higher error (RSME) with statistical features compared with the wavelet-based advanced features emphasized the need for sophisticated features to predict RGC count accurately. SVM- and GPR-based models provided the most accurate prediction of RGC numbers from ERG signals. Specifically, squared exponential and rational quadratic models of GPR provided the least error.

**Table 4:** Performance metrics for retinal ganglion cells (RGCs) Regression.

**Machine learning algorithm**	**RSME**	
	**Statistical**	**Wavelet**
Tree	31.716	17.852
SVM	17.177	13.82
Ensemble (Bagged)	29.129	24.387
Logistic regression	44.622	24.873
**Gaussian process regression**	**15.644**	**11.201**

*Bold font indicate the best performing regression model and its corresponding RSME*.

## 4. Discussion

Our goal was to determine the feasibility of applying ML-based methods to the analysis of ERG signals for glaucoma detection at different stages of the disease. In the present study, we systematically applied machine-learning-based methods for the first time to detect glaucoma and predict RGC loss based on ERG signals. The present study utilized ERGs measured in mice rather than from human patients, because the use of data from a preclinical model allowed us to validate “ground truth” data sets with a range of complimentary and alternative experimental strategies, which is not possible in human clinical studies. These include histology, biochemical, and immunochemical assays, as well as optomotor reflex measurements. We were able to determine for the first time that advanced features (wavelet-based features) are capable of detecting subtle changes in the ERG signal and perform multiclass classification based on the progression level of the disease with 80% accuracy. In particular, we found that Shannon Entropy Values for Maximal Overlap Discrete Wavelet Packet Transform (MOD-PWT) and AR coefficients represent important features capable of detecting early-stage glaucoma. Among the nine available ERG signals, Flicker, STR, OP, and Rod-Cone appear integral for such successful detection. This is in agreement with the results published in Lei et al. (2006). However, given that these features are highly correlated, the ML-based algorithm picks only one for each set of highly correlated features to reduce the model complexity as shown in Figure 6.

In addition, the method proposed here performs ERG analysis in a wavelet domain instead of a frequency domain, which allows to capture subtle changes in the signals. In addition, various intricate features such as multiscale wavelet variance estimates, Shannon entropy, and autoregressive coefficients are incorporated in the method, compared to basic features such as differences in amplitude and latency in previous studies (Hood et al., 2000; Fortune et al., 2002; Thienprasiddhi et al., 2003; Stiefelmeyer et al., 2004; Ventura and Porciatti, 2006; Chu et al., 2007; Miguel-Jiménez et al., 2010; Luo et al., 2011; Palmowski-Wolfe et al., 2011; Todorova and Palmowski-Wolfe, 2011; Ho et al., 2012; Hori et al., 2012; Ledolter et al., 2013; Consejo et al., 2019). The results strongly suggest that such advanced features in the wavelet domain are necessary for detection of early-stage glaucoma. Moreover, in contrast to the recent study that leverages ML-based technique to analyze ERG using solely the photopic negative response (PhNR) component (Armstrong and Lorch, 2020), the current method uses all ERG components in the analysis to fully utilize the capability of the ML-based technique to crunch large data sets and draw complicated relationships. Therefore, the proposed framework is not limited to a small subset of genetic eye diseases like previous studies (Fortune et al., 2002; Thienprasiddhi et al., 2003; Stiefelmeyer et al., 2004; Chu et al., 2007; Miguel-Jiménez et al., 2010; Luo et al., 2011; Palmowski-Wolfe et al., 2011; Todorova and Palmowski-Wolfe, 2011; Ho et al., 2012; Hori et al., 2012; Ledolter et al., 2013; Consejo et al., 2019); instead, it is capable of mapping ERG signals to various eye diseases.

## 5. Conclusion

Results obtained in the present study strongly suggest that the methods employed can reproducibly identify dominant features for classification and regression from STR, Oscillatory potentials (OPs), and other ERG tests consistent with the results reported in previously published work on the sensitivity of and OPs and flicker to subtle changes in RGC function and viability (Tyler, 1981; Brandao et al., 2017). Further, our approach identified additional dominant distinguishing features such as Shannon Entropy Values for Maximal Overlap Discrete Wavelet Packet Transform (MOD-PWT) and AR coefficients, which are not distinguishable by traditional methods used in Grillo et al. (2018). This strongly suggests that the current machine-learning-based algorithm has significant potential in distinguishing subtle changes in ERG signals corresponding to different stages of glaucoma disease development. This capability of the technique could be used as a foundational step to create a reliable framework for the early detection of glaucoma and to monitor efficacy of therapeutic intervention in both clinical practice and novel drug development for glaucoma. In addition, the inclusion of various ERG protocols in this framework, such as cones, rods and cones, STR, and oscillatory potentials, represent responses from different cell types in the eye. Therefore, ERG response can be mapped to diseases specific to those cell types. It should be noted that this study was based on mice and with 12 h of dark adaptation. The promising results obtained here suggest the great potential for this method to help detect early stage, pre-symptomatic glaucoma. However, an additional study on adaptation requirements would be required before extending this framework to humans.

## Data Availability Statement

The datasets generated for this study are available on request to the corresponding author. Requests to access these datasets should be directed to mehdizadeha@umkc.edu.

## Author Contributions

MG contributed in machine learning framework development, formal analysis, investigation, validation, visualization, and writing—original draft. LR contributed in writing—review and editing. PK contributed in providing the data, conceptualization, supervision, and writing—review and editing. AM contributed in conceptualization, supervision, and writing—review and editing. All authors contributed to the article and approved the submitted version.

## Funding

Research reported in this publication was supported by the Felix and Carmen Sabates Missouri Endowed Chair in Vision Research, the Vision Research Foundation of Kansas City, and in part by National Eye Institute grant EY031248 of the National Institutes of Health (PK). The content is solely the responsibility of the authors and does not necessarily represent the official views of the National Institutes of Health. The publication cost was covered by PK.

## Author Disclaimer

The content is solely the responsibility of the authors and does not necessarily represent the official views of the National Institutes of Health.

## Conflict of Interest

PK, AM, and MG have a patent-pending based on this study. The remaining author declares that the research was conducted in the absence of any commercial or financial relationships that could be construed as a potential conflict of interest.

## Publisher's Note

All claims expressed in this article are solely those of the authors and do not necessarily represent those of their affiliated organizations, or those of the publisher, the editors and the reviewers. Any product that may be evaluated in this article, or claim that may be made by its manufacturer, is not guaranteed or endorsed by the publisher.
